# Histological Characterization of Biliary Intraepithelial Neoplasia with respect to Pancreatic Intraepithelial Neoplasia

**DOI:** 10.1155/2014/678260

**Published:** 2014-04-10

**Authors:** Yasunori Sato, Kenichi Harada, Motoko Sasaki, Yasuni Nakanuma

**Affiliations:** ^1^Department of Human Pathology, Kanazawa University Graduate School of Medicine, 13-1 Takara-machi, Kanazawa 920-8640, Japan; ^2^Department of Pathology, Shizuoka Cancer Center, 1007 Shimonagakubo, Nagaizumi-cho, Sunto-gun, Shizuoka 411-8777, Japan

## Abstract

Biliary intraepithelial neoplasia (BilIN) is a precursor lesion of hilar/perihilar and extrahepatic cholangiocarcinoma. BilIN represents the process of multistep cholangiocarcinogenesis and is the biliary counterpart of pancreatic intraepithelial neoplasia (PanIN). This study was performed to clarify the histological characteristics of BilIN in relation to PanIN. Using paraffin-embedded tissue sections of surgically resected specimens of cholangiocarcinoma associated with BilIN and pancreatic ductal adenocarcinoma associated with PanIN, immunohistochemical staining was performed using primary antibodies against MUC1, MUC2, MUC5AC, cyclin D1, p21, p53, and S100P. For mucin staining, Alcian blue pH 2.5 was used. Most of the molecules examined here showed similar expression patterns in BilIN and PanIN, in which their expression tended to increase along with the increase in atypia of the epithelial lesions. Significant differences were observed in the increase in mucin production and the expression of S100P in PanIN-1 and the expression of p53 in PanIN-3, when compared with those in BilIN of a corresponding grade. These results suggest that cholangiocarcinoma and pancreatic ductal adenocarcinoma share, at least in part, a common carcinogenic process and further confirm that BilIN can be regarded as the biliary counterpart of PanIN.

## 1. Introduction

Cholangiocarcinoma that arises under conditions of chronic biliary diseases such as hepatolithiasis often undergoes the multistep carcinogenesis process [1]. Biliary intraepithelial neoplasia (BilIN) is known as a premalignant lesion of cholangiocarcinoma that represents the multistep cholangiocarcinogenesis [2]. The classification is applicable to flat atypical epithelial lesions in the intrahepatic large bile ducts and the extrahepatic bile ducts, and it is also applied to lesions in the gallbladder according to the current World Health Organization (WHO) classification for tumors of the digestive system [3].

BilIN is a concept that is proposed based on the morphological resemblance to pancreatic intraepithelial neoplasia (PanIN). Similar to PanIN, BilIN is classified into three grades according to the degree of cytological and architectural atypia: BilIN-1 (low-grade lesions), BilIN-2 (intermediate-grade lesions), and BilIN-3 (high-grade lesions, carcinoma in situ). Using the BilIN classification, there is increasing evidence that molecular and genetic alterations accumulate during the progression of BilIN to cholangiocarcinoma [4–7].

Since the biliary tract and pancreas share a common developmental process as well as morphological characteristics as duct systems, it is plausible that some biliary and pancreatic diseases show similar pathological features and biological behaviors [8]. Indeed, our recent comparative analysis showed that hilar cholangiocarcinoma and ductal adenocarcinoma of the pancreas share many clinicopathological features [9]. In addition, we showed that intraductal papillary neoplasm of the bile duct (IPNB) and intraductal papillary mucinous neoplasm (IPMN) of the pancreas, as well as mucinous cystic neoplasm (MCN) of the biliary tract and pancreas, exhibit similar immunohistochemical phenotypes, suggesting a common carcinogenic process of the tumors [10], where all these tumors were classified as premalignant lesions according to the current WHO classification.

As far as the histological characteristics of BilIN and PanIN are concerned, previous studies have examined their features individually, and detailed data on comparative analysis of BilIN and PanIN are lacking. This study was therefore conducted to clarify the histological characteristics of BilIN with respect to PanIN.

## 2. Materials and Methods

### 2.1. Tissue Preparation

Hepatolithiatic livers associated with perihilar cholangiocarcinoma were used as a model of multistep cholangiocarcinogenesis. A total of 25 hepatolithiatic livers with cholangiocarcinoma and a total of 22 pancreatic specimens with pancreatic ductal adenocarcinoma were retrieved from the files of our laboratory and affiliated hospitals. The patients were selected during the period between 1997 and 2007. All cases were surgically resected, and all liver and pancreatic specimens were histologically accompanied by BilIN and PanIN, respectively. In all cases of cholangiocarcinoma, the main part of the tumor was located in hilar or perihilar region of the liver, and they appeared to arise from the intrahepatic large bile ducts or the right or left hepatic bile duct. Most cholangiocarcinoma cases showed macroscopic features of mass-forming type and/or intraductal growth type. Foci of BilIN were microscopically located in the intrahepatic large bile ducts and the hepatic bile ducts, and they were not observed in the septal and interlobular bile ducts. The mean age and sex distribution (male-female ratio) of the patients were 62 years and 11 : 14 for the liver specimens and 68 years and 12 : 10 for the pancreatic specimens, respectively. The samples were fixed in 10% neutral formalin and embedded in paraffin. Then, 4-*μ*m-thick paraffin-embedded sections were prepared. One representative section from each case was used.

### 2.2. Histochemistry and Immunohistochemistry

Alcian blue (at pH 2.5) was used for mucin staining. Immunostaining was performed using the sections with the primary antibodies listed in Table 1. After the blocking of endogenous peroxidase, the sections were incubated in protein block solution (DakoCytomation, Glostrup, Denmark). They were then incubated overnight at 4°C with each of the primary antibodies. Their sources, optimal dilution, and antigen retrieval methods are shown in Table 1. They were treated with secondary antibodies conjugated to a peroxidase-labeled polymer using the HISTOFINE system (Nichirei, Tokyo, Japan). Color development was performed using 3,3′-diaminobenzidine tetrahydrochloride, and the sections were lightly counterstained with hematoxylin. Negative controls consisted of substitution of the primary antibodies with nonimmune serum and were consistently negative.

### 2.3. Histological Assessment

Semiquantitative analysis of the stained sections was performed. Staining intensity was evaluated in a high-power field for the neoplastic and nonneoplastic epithelia of the bile ducts and pancreatic ducts. From the sections of 25 liver specimens and 22 pancreatic specimens, foci of interest were selected. The number of foci examined was as follows: nonneoplastic large bile duct, 14; BilIN-1, 17; BilIN-2/3, 24, invasive carcinoma (cholangiocarcinoma), 50; nonneoplastic pancreatic duct, 13; PanIN-1, 22; PanIN-2/3, 15; invasive carcinoma (pancreatic ductal adenocarcinoma), 44.

For mucin staining with Alcian blue (pH 2.5), the signal intensity in the cytoplasm and/or on the luminal surface of the epithelial cells was evaluated using the following grading system: 1+ (mild), 2+ (moderate), and 3+ (marked). The cytoplasmic and/or luminal immunostaining of MUC1 and the cytoplasmic immunostaining of MUC2 and MUC5AC were graded as follows: 0 (negative), 1+ (mild to moderate), and 2+ (marked). For evaluation of the nuclear staining of cyclin D1, p21, p53, and S100P, the percentage of positive nuclei to the total number of nuclei of the epithelial cells was calculated, and it was graded as follows: 0 (negative), 1+ (not exceeding 10%), and 2+ (more than 10%). For p53 nuclear staining, only the proportion of intensely positive nuclei was scored.

### 2.4. Statistics

Statistical significance was determined using the Mann-Whitney *U*-test. A *P* value less than 0.05 was accepted as the level of statistical significance.

## 3. Results and Discussion

**Figure 1:**
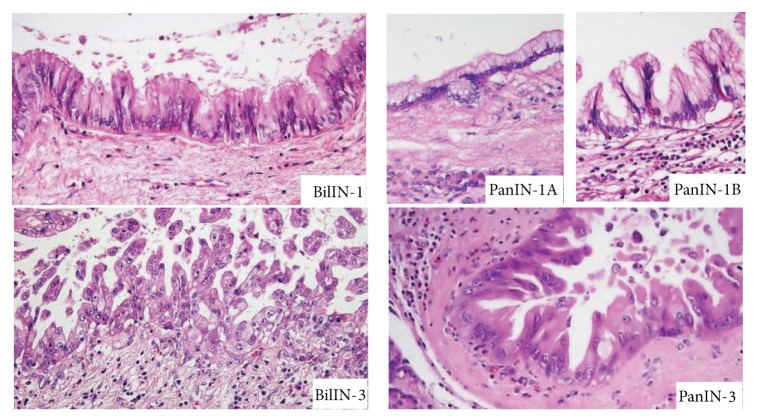
Histology of biliary intraepithelial neoplasia (BilIN) and pancreatic intraepithelial neoplasia (PanIN). Representative images of BilIN-1 and BilIN-3 and PanIN-1A, PanIN-1B, and PanIN-3 are shown. Hematoxylin and eosin staining. Original magnifications, ×400.

Morphological appearances such as loss of nuclear polarity, increased nucleus-to-cytoplasm ratio, nuclear hyperchromasia, and architectural atypia were basically similar between the corresponding grades of BilIN and PanIN, which were observed in sections stained with hematoxylin and eosin (Figure 1).

**Figure 2:**
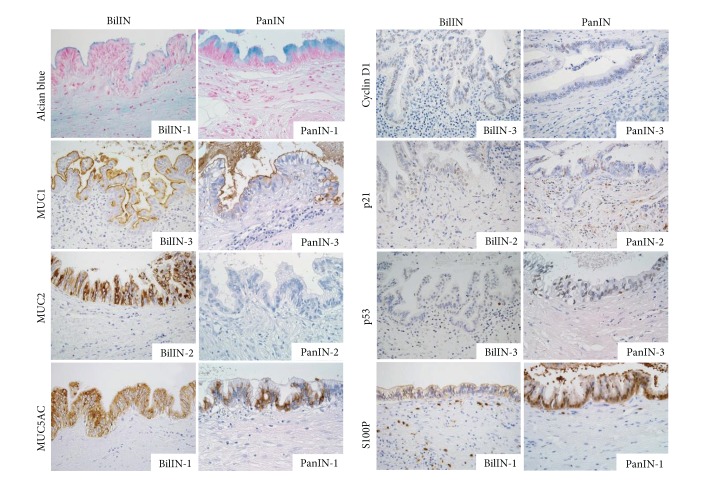
Representative images of histochemical and immunohistochemical staining. The results of mucin staining with Alcian blue (pH 2.5) and immunostaining of MUC1, MUC2, MUC5AC, cyclin D1, p21, p53, and S100P for biliary intraepithelial neoplasia (BilIN) and pancreatic intraepithelial neoplasia (PanIN) are shown. Original magnifications, ×400.

**Figure 3:**
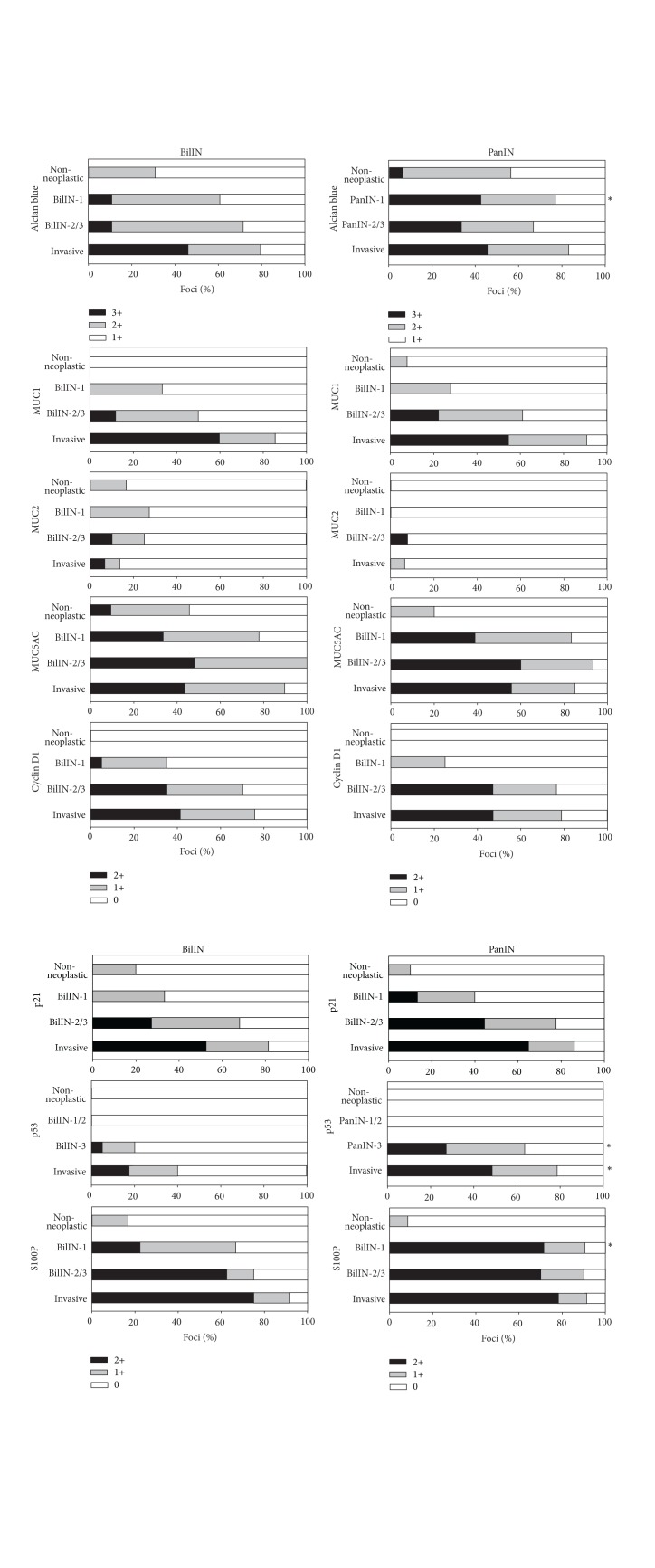
Semiquantitative analysis of the results of histochemical and immunohistochemical staining. The analysis was performed as described in Section 2 for the lesions of nonneoplastic epithelium of the bile ducts and the pancreatic ducts, biliary intraepithelial neoplasia (BilIN), pancreatic intraepithelial neoplasia (PanIN), and invasive carcinoma. *P < 0.05 versus the results of BilIN of corresponding histological grade or cholangiocarcinoma.

Mucin staining with Alcian blue (pH 2.5) showed that both BilIN and PanIN frequently had cytoplasmic and/or luminal surface mucin (Figure 2). According to the grade of BilIN and PanIN, PanIN-1 tended to have more abundant cytoplasmic mucin than BilIN-1, and the results of semiquantitative analysis confirmed this tendency (Figure 3). The abundant mucin expression in PanIN-1 is consistent with the definition of PanIN-1 in which the lesion is composed of tall columnar cells with basally located nuclei and abundant supranuclear mucin [11].

The immunohistochemical expression of MUC1 was increased along with the increase in the grade of BilIN and PanIN, and no significant difference in its expression status was observed between BilIN and PanIN (Figures 2 and 3). Similarly, the expression of MUC5AC was frequently observed in all grades of both BilIN and PanIN (Figures 2 and 3). The results of the expression status of MUC1 and MUC5AC in BilIN were almost identical to those in our previous report [4].

Focal immunohistochemical expression of MUC2 was observed in several foci of BilIN, whereas MUC2 positivity was exceptional in PanIN (Figures 2 and 3). Although the expression of CK20 was typically negative in both BilIN and PanIN in this study (data not shown), BilIN is not infrequently associated with metaplastic change of intestinal type, while intestinal-type PanIN is generally not found [12, 13]. These observations may explain the focal MUC2 expression in BilIN rather than in PanIN.

The results of immunostaining of MUC1, MUC2, MUC5AC, and CK20 for BilIN and PanIN in this study are summarized in Table 2. For comparison, the results of our previous comparative analysis that examined the immunohistochemical characteristics of IPNB, IPMN of the pancreas, hepatic MCN, and pancreatic MCN [10] are also shown in Table 2. It is noteworthy that all of these premalignant lesions show similar immunoprofiles to each other between the biliary tract and pancreas, supporting the concept that BilIN, IPNB, and hepatic MCN are biliary counterparts of PanIN, IPMN, and pancreatic MCN, respectively.

As for the expression of cell cycle-related molecules, the immunohistochemical expression of cyclin D1 and p21 was absent or focal in nonneoplastic epithelium of the bile ducts and the pancreatic ducts. They were occasionally observed in BilIN-1 and PanIN-1 and more frequently in BilIN-2/3 and PanIN-2/3 (Figures 2 and 3), in which the frequency of the expression of cyclin D1 and p21 in BilIN in this study was comparable to that in our previous report [5]. Semiquantitative analysis showed that there was no significant difference in their expression status between BilIN and PanIN.

The expression of p53 was not observed in nonneoplastic epithelium of the bile ducts and the pancreatic ducts, as well as in BilIN-1/2 and PanIN-1/2. By contrast, BilIN-3 and PanIN-3 occasionally showed the expression of p53, and its frequency was significantly higher in PanIN-3 than in BilIN-3 (Figures 2 and 3). Because the process of carcinogenesis is often complicated by inflammatory changes in the biliary tract, the molecular alterations may be more complex in BilIN due to cholangitis than those seen in PanIN, where the influence of inflammation is usually insignificant in the development of pancreatic cancer. In fact, our recent study showed that the detection rate of* KRAS* mutation in BilIN was not as high as that seen in PanIN [6]. Therefore, it is predicted that factors other than genetic alterations may also affect the process of the development of BilIN and cholangiocarcinoma.

S100P is a molecule that is highly expressed by perihilar and extrahepatic cholangiocarcinoma as well as pancreatic ductal adenocarcinoma [9, 14]. In this study, the expression of S100P was frequently observed in both BilIN and PanIN of all grades (Figure 2). Semiquantitative analysis showed that its expression was significantly high in PanIN-1 compared with that in BilIN-1, although both BilIN-1 and PanIN-1 exhibited a high frequency of S100P expression (Figure 3).

Most of the molecules examined in this study showed similar expression patterns in BilIN and PanIN. There were significant differences in the increase in mucin production and the expression of S100P in PanIN-1 and the expression of p53 in PanIN-3, when compared with those in BilIN of corresponding grade.

The immunohistochemical expression of MUC1, cyclin D1, p21, p53, and S100P tended to be increased in invasive foci of cholangiocarcinoma and pancreatic ductal adenocarcinoma when compared to those in BilIN-2/3 and PanIN-2/3, respectively (Figure 3). These results were consistent with the concept of multistep carcinogenesis.

## 4. Conclusions

BilIN and PanIN showed similar histological and immunohistochemical features with several exceptions. These results suggest that cholangiocarcinoma and pancreatic ductal adenocarcinoma share, at least in part, a common carcinogenic process and further confirm that BilIN can be regarded as the biliary counterpart of PanIN.

## Figures and Tables

**Table 1:** Primary antibodies used for immunohistochemical analysis.

Antigen	Clone	Company	Dilution	Antigen retrieval
MUC1	DF3	Toray Fuji Bionics (Tokyo, Japan)	1 : 50	MW
MUC2	Ccp58	Novocastra (Newcastle, UK)	1 : 100	MW
MUC5AC	CLH2	Novocastra	1 : 200	MW
Cytokeratin 20	Ks 20.8	DakoCytomation (Glostrup, Denmark)	1 : 50	MW
Cyclin D1	SP4	Nichirei (Tokyo, Japan)	Prediluted	MW*
p21	EPR3993	Abcam (Cambridge, UK)	1 : 100	MW
p53	DO-7	DakoCytomation	1 : 100	MW
S100P	EPR6143	Abcam	1 : 100	MW

MW: microwaving in 10 nmol/L citrate buffer (pH 6.0) for 20 minutes; MW*: microwaving in tris-ethylenediaminetetraacetic acid buffer (pH 9.0) for 20 minutes.

**Table 2:** Immunoprofiles of premalignant lesions of the biliary tract and pancreas.

	Intraepithelial neoplasia		Intraductal papillary neoplasm		Mucinous cystic neoplasm	
	BilIN	PanIN	IPNB	IPMN	Hepatic MCN	Pancreatic MCN
MUC1	+	+	+	+	+	+
MUC2	+	−	+	+	−	−
MUC5AC	++	++	++	++	++	++
CK20	−	−	+	+	−	−

The results of comparative analysis for biliary and pancreatic neoplasms in the present study and our previous report (10) are summarized. −: likely absent; +: occasionally present; ++: usually present. BilIN: biliary intraepithelial neoplasia; CK: cytokeratin; IPMN: intraductal papillary mucinous neoplasm; IPNB: intraductal papillary neoplasm of the bile duct; MCN: mucinous cystic neoplasm; PanIN: pancreatic intraepithelial neoplasia.

## Abbreviations

BilIN: Biliary intraepithelial neoplasia
CK: Cytokeratin
IPMN: Intraductal papillary mucinous neoplasm
IPNB: Intraductal papillary neoplasm of the bile duct
MCN: Mucinous cystic neoplasm
PanIN: Pancreatic intraepithelial neoplasia
WHO: World Health Organization.
